# Building quality primary health care development in the new era towards universal health coverage: a Beijing initiative

**DOI:** 10.1186/s41256-023-00341-y

**Published:** 2023-12-18

**Authors:** Minghui Ren, Tuohong Zhang, Jin Xu, Jie Qiao, Jianrong Qiao, Siyan Zhan, Jiangmei Qin, Daping Song, Yanru Fang, Yifang Lin, Xiaopeng Jiang, Yan Guo, Qingyue Meng, Xu Qian, Yunguo Liu, Sophia Siu Chee Chan, Feng Zhao, Winnie Yip, Hong Wang, Minmin Wang, Hui Yin, Zuokun Liu, Na Li, Xinyi Song, Fangfang Liu, Yinzi Jin, Fangjing Liu, Yangmu Huang

**Affiliations:** 1 Department of Global Health, School of Public Health, Peking University, Beijing, China; 2 Institute for Global Health, Peking University, Beijing, China; 3 China Centre for Health Development Studies, Peking University, Beijing, China; 4 Center for Reproductive Medicine, Department of Obstetrics and Gynecology, Peking University Third Hospital, Beijing, China; 5 World Health Organization Representative Office in China, Beijing, China; 6 Department of Epidemiology and Biostatistics, School of Public Health, Peking University Health Science Center, Beijing, China; 7 China National Health Development Research Center, Beijing, China; 8 College of Agronomy and Biotechnology, China Agricultural University, Beijing, China; 9 Department of Rehabilitation Medicine, Huashan Hospital, Fudan University, Shanghai, China; 10 School of Public Health, Fudan University, Shanghai, China; 11 Global Health Research Center, Duke Kunshan University, Kunshan, Jiangsu China; 12 School of Nursing, LKS Faculty of Medicine, The University of Hong Kong, Pok Fu Lam, Hong Kong SAR China; 13 Health Nutrition and Population, South Asia Region, World Bank, Bangkok, Thailand; 14 Department of Global Health and Population, Harvard T H Chan School of Public Health, Boston, MA USA; 15 Bill & Melinda Gates Foundation, Beijing, China

**Keywords:** Primary health care, Universal health coverage, Sustainable development agenda

## Abstract

Primary health care (PHC) is the most effective way to improve people's health and well-being, and primary care services should act as the cornerstone of a resilient health system and the foundation of universal health coverage. To promote high quality development of PHC, an International Symposium on Quality Primary Health Care Development was held on December 4–5, 2023 in Beijing, China, and the participants have proposed and advocated the Beijing Initiative on Quality Primary Health Care Development. The Beijing Initiative calls on all countries to carry out and strengthen 11 actions: fulfill political commitment and accountability; achieve “health in all policies” through multisectoral coordination; establish sustainable financing; empower communities and individuals; provide community-based integrated care; promote the connection and integration of health services and social services through good governance; enhance training, allocation and motivation of health workforce, and medical education; expand application of traditional and alternative medicine for disease prevention and illness healing; empower PHC with digital technology; ensure access to medicinal products and appropriate technologies; and last, strengthen global partnership and international health cooperation. The Initiative will enrich the content of quality development of PHC, build consensus, and put forward policies for quality development of PHC in China in the new era, which are expected to make contributions in accelerating global actions.

The importance of primary health care (PHC) is particularly evident as human beings experience profound challenges, including social transitions and the pandemic caused by infectious diseases[1]. PHC has its unique advantage in addressing challenges to global health posed by ageing and climate change, prevailing and constantly increasing burden of non-communicable diseases and mental health, and the "last-mile" of access to health care and medical products[2, 3].

Since the adoption of the Alma Ata Declaration[4], countries around the world have accumulated valuable experience in implementing PHC and improving people's health across diverse socioeconomic settings. Such experience constitutes a common treasure of human beings and an important foundation for achieving the health-related 2030 Sustainable Development Goals[5, 6]. China is one of the earliest countries in the world providing the model of community-led PHC. China's three-level health service network, barefoot doctors, cooperative medical scheme, and integration of traditional Chinese and Western medicine was concluded by the World Health Organization and the United Nations Children's Fund as appropriate workforce and appropriate technology. With coordinated and continuous effort such as the patriotic health campaign, the implementation of health system reform and Healthy China 2030 strategy, China continues to adhere to the approach of PHC in practice and firmly supports the promotion of PHC at all stages of socioeconomic development.

We believe that PHC should be quality assured. By quality, we mean PHC should be people-centered, integrated, comprehensive, continuous, and embedded in the community; it should be accessible, affordable, and acceptable to all people; primary care services should be provided by well-trained, competent, effectively motivated, and highly committed health personnel; primary care services should act as the cornerstone of a resilient health system and the foundation of universal health coverage (UHC)[7, 8]. And more importantly, it should continue to adapt and develop, in particular taking advantage of technological advancement. Building quality PHC is the shared responsibility of all government departments and all sectors of society.

To promote high quality development of PHC, an International Symposium on Quality PHC Development was held on December 4–5, 2023 in Beijing, China, hosted by Peking University Health Science Center, organized by Peking University School of Public Health and Peking University Institute for Global Health, with supports from Department of Primary Health of China’s National Health Commission, and World Health Organization (WHO) Representative Office in China. More than 200 leaders and experts from government departments, academic institutions, as well as World Health Organization, United Nations Children's Fund, Bill and Melinda Gates Foundation, and World Organization of Family Doctors (WONCA) in China, Spain, New Zealand, Republic of Korea, Mongolia, Hong Kong Special Administrative Region (SAR), Macao SAR and other countries and regions, attended this international symposium.

**Figure:**
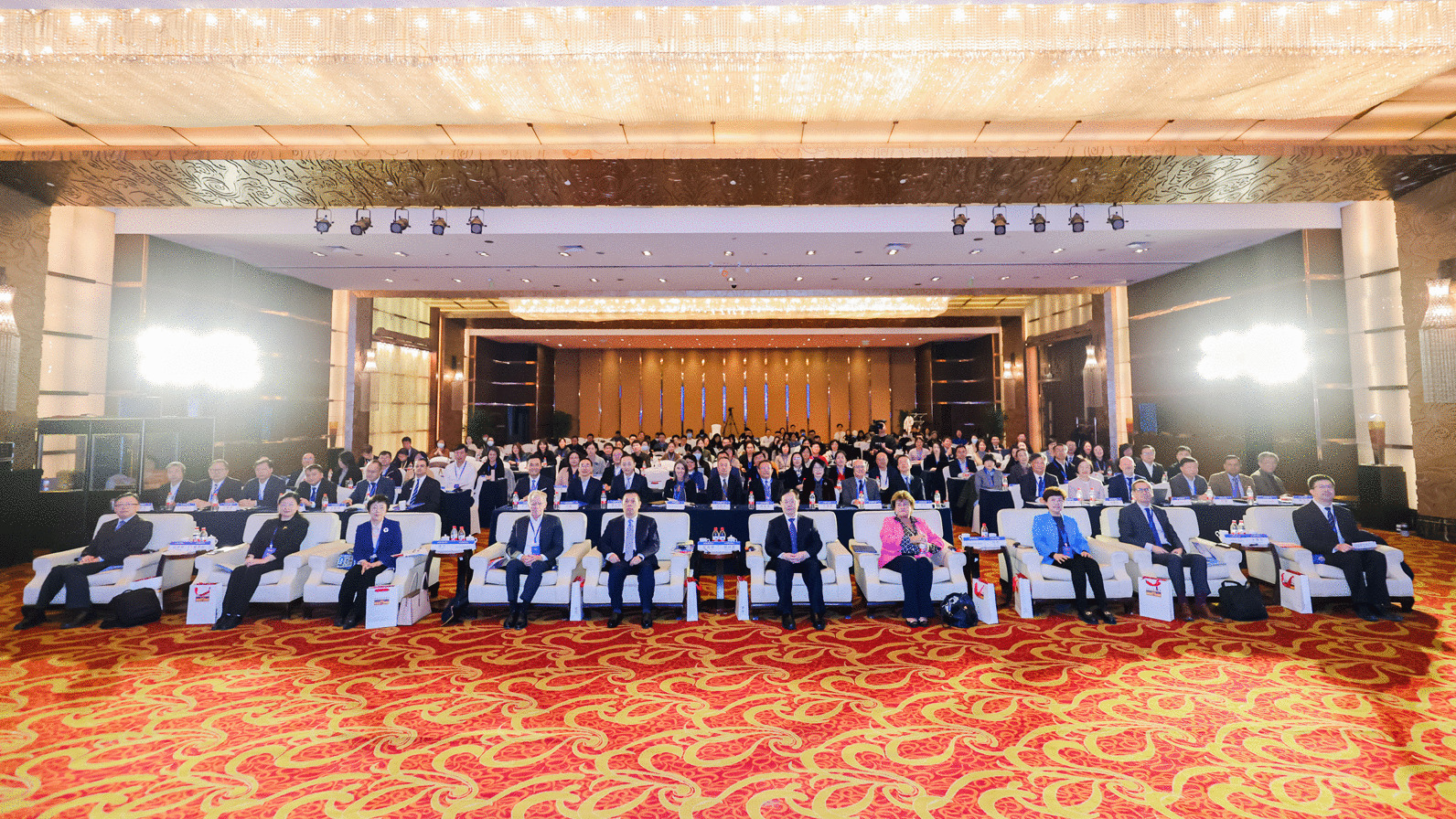
(Copy right © International Symposium on Quality Primary Health Care Development).

The participants of the symposium unanimously agree with and insisting the principles of the Declaration of Alma-Ata[4] and the Declaration of Astana[9] that PHC is the most effective way to improve people's health and well-being. The United Nations’ Sustainable Development Goals and the Political Declaration of the United Nations General Assembly’s 2023 high-level meeting on Universal Health Coverage[10] have reaffirmed the importance of PHC in our time. The participants reemphasize that in all countries and regions, the approach of PHC is not only valid but also critical. The Beijing Initiative on Quality Primary Health Care Development is proposed, which calls on all countries to carry out and strengthen the following 11 actions:

**Figure:**
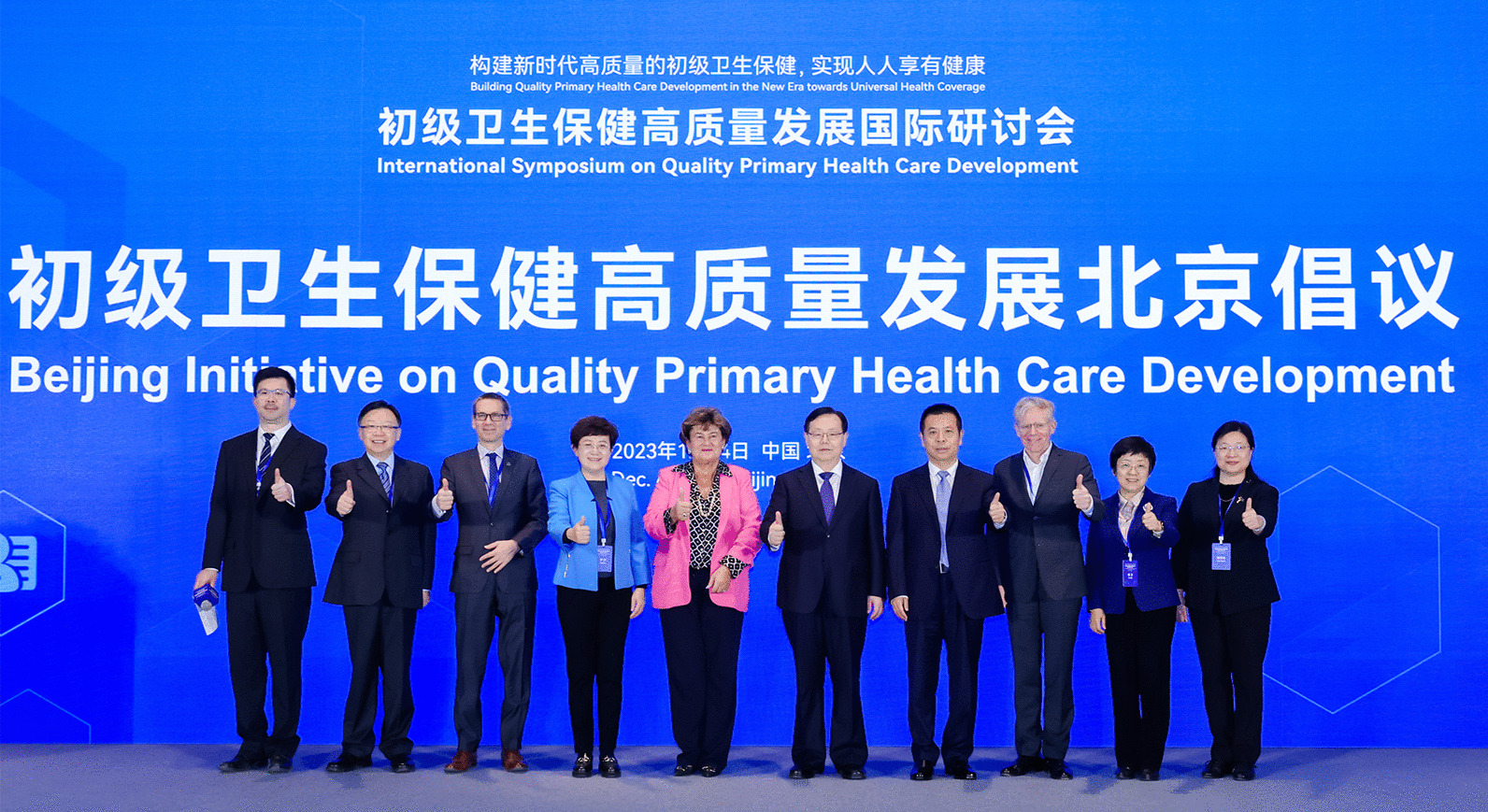
(Copy right © International Symposium on Quality Primary Health Care Development).

## Fulfill political commitment and accountability

We call on all political leaders and governments to uphold the goal of improving the health and well-being of individuals and populations, fulfill their responsibilities in leadership, safeguarding and supervision, and steward health development and reform, to build a strong and high-quality PHC system as part of integrated people-centered health services. All political leaders and governments should put prevention first and integrate prevention and treatment, promote equal opportunities for all people to enjoy accessible and affordable essential health services, and promote high quality development of PHC.

## Achieve "health in all policies (HiAP)" through multisectoral coordination

We advocate the approach of "health in all policies (HiAP)" and emphasize the important role of public policies for public health. All sectors and industries should strengthen communication and collaboration, integrate health into various policies and their formulation processes, and form synergy to promote health. We support the establishment of a system of health impact assessment and evaluation, improve the mechanism of supervision and accountability, and ensure that HiAP is implemented and enforced.

## Establish sustainable financing

We advocate the establishment of a sustainable financing strategy for PHC and increasing and ensuring adequate public funding. We advocate the establishment of an equity-oriented financing mechanism, specification of a primary health service package that can be expanded over time and adapted to national and local context, coordination of multi-channel health funding for disease prevention and treatment and ensuring that funding for primary care facilities and front-line service providers are fully paid in their budgets. We call for strategic purchasing and step-wise shift towards a value-oriented blended payment method with capitation as the base. We call for using health insurance funds to purchase preventive services. We call for provision of sustainable financing support to low-income countries for development in PHC.

## Empower communities and individuals

We call on raising health awareness to ensure that everyone participates in, makes their best contribution to, and enjoys health improvement. We advocate communities to create a healthy ecosystem and living environment that benefits the entire population horizontally and covers the entire life cycle vertically. We call for a focus on prevention to reduce the occurrence of diseases. We advocate provision of accessible health services in the community, to achieve early diagnosis, early treatment, and early rehabilitation of diseases and health conditions.

## Provide community-based integrated care

We advocate the establishment of community-based integrated primary health services that cover the entire population and meet the daily health needs of people of all ages and from all population groups. We advocate the establishment of primary care services as the core, to coordinate all-round health services for patients and community residents and achieve the continuity and integration of prevention, treatment, nursing, rehabilitation and palliative care across all settings (including families, living communities, health care institutions, and long-term care institutions), exploration of innovative health service models to integrate medical treatment and prevention. We call upon integration of PHC into the core components in preparedness, prevention and response to pandemic and other public health emergencies.

## Promote the connection and integration of health services and social services through good governance

We call for good health governance functions and better alignment between implementation and policies, to empower and promote community engagement and participation. We call for strengthening community-level governance, enhancing the connection between PHC services and social services, and promoting the improvement of population health. Within the community catchment, governments, autonomous organizations and others should play fully the role of resource allocation, enhance hub function of primary care providers such as health coordinators and family doctor teams, improve health literacy, strengthen the construction of self-management groups, and cooperate with civil societies, volunteers, and economic organizations, to deeply integrate public services such as elderly care, disability assistance, and mental health, psychological counseling into primary care services.

## Enhance training, allocation and motivation of health workforce, and medical education

We support increasing allocation of health workforce in rural, remote and underdeveloped areas, promoting equity in human resource allocation, and strengthening PHC services with appropriate human resources allocation. The training of general practitioners/family doctors should be strengthened, and an incentive mechanism for the training and employment of general practitioners/family doctors should be established. We call for creating a good working environment for health personnel working in PHC settings, providing reasonable remuneration, to motivate providers to effectively meet the basic health service needs of people.

## Expand application of traditional and alternative medicine for disease prevention and illness healing

We recognize traditional medicine as an integral part of PHC, and support the application of appropriate technologies of traditional medicine and alternative medicine to the community. We support that primary care institutions should provide effective alternative medicine services from prevention and health care to treatment and rehabilitation. We support that people voluntarily use traditional and alternative medicine to prevent and treat diseases, under the guidance of community health services professionals.

## Empower PHC with digital technology

We advocate strengthened application of digital technology in PHC, to improve the quality, individualization, precision and convenience of PHC services. We advocate promotion of the exchange and sharing of health information with residents' electronic health records as the core. We advocate development of artificial intelligence-assisted diagnosis, telemedicine and other means to help improve the quality of diagnosis and treatment. We call for further development of online appointment booking, cloud-based consultation, electronic prescription, smart pharmacy, online payment, remote health education and other applications and wearable health devices to facilitate comprehensive health management and self-management of health. With rapid scale up, we call for effective governance and regulation to harness digital innovation to minimize risks while maximizing benefits.

## Ensure access to medicinal products and appropriate technologies

We emphasize the key role of PHC in ensuring that medicines, vaccines, appropriate technologies, are accessible to the entire population, especially the vulnerable groups. We call for further integration of the pharmaceutical product supply system and the PHC system; we support primary health service providers to better serve the equitable accessibility of pharmaceutical products throughout the life cycle and appropriate technology applications, and exploration of the innovation and promotion of integrated PHC, such as prenatal care packages and nutrition packages.

## Strengthen global partnership and international health cooperation

We call for the establishment of multi-level and multi-form international cooperation and exchange channels, strengthening of global partnerships on PHC, and pragmatic cooperation in experience exchange, technology promotion, personnel training, health assistance, resource mobilization; maintaining, improving, and promoting the health of all population groups, in particular children, adolescents, pregnant women, the elderly, disabled people, the poor and other vulnerable groups, effectively prevent, manage, and control communicable diseases, non-communicable diseases, and other major health challenges, leaving no one behind and promoting UHC.

## Data Availability

Data sharing not applicable to this article as no datasets were generated or analyzed during the current study.

## Abbreviations

HiAP: Health in all policies
PHC: Primary health care
SAR: Special administrative region
WHO: World health organization
WONCA: World organization of family doctors
UHC: Universal health coverage
